# Parallel analysis of multiple human memory CD4 ^+^ T‐cell subsets within antigen‐specific responses using cell proliferation dyes

**DOI:** 10.1111/imcb.12606

**Published:** 2022-11-28

**Authors:** Laura Cook, John Zaunders, Nabila Seddiki, David van Bockel, Anthony D Kelleher, C. Mee Ling Munier

**Affiliations:** ^1^ Immunovirology and Pathogenesis Program The Kirby Institute, UNSW Sydney NSW Australia; ^2^ St Vincent's Centre for Applied Medical Research, St Vincent's Hospital Sydney NSW Australia; ^3^ Present address: Department of Microbiology and Immunology University of Melbourne, at The Peter Doherty Institute for Infection and Immunity Parkville VIC 3000 Australia; ^4^ Present address: IDMIT Department/IBFJ, Immunology of Viral Infections and Autoimmune Diseases (IMVA), INSERM U1184, CEA Université Paris Sud Paris France

**Keywords:** AIM assay, antigen‐specific response, cell proliferation, memory, Tregs

## Abstract

Activation induced marker (AIM) assays are being used increasingly to measure antigen‐specific T‐cell responses, but this activation can alter cell lineage defining phenotypic markers. We aimed to extend the utility of AIM assays to enable pre‐activation defined cell populations to be tracked and quantified within T‐cell memory responses. We sorted three *ex vivo* CD4^+^ T‐cell populations prior to any activation using well defined *ex vivo* lineage surface marker combinations. These populations were memory non‐Tregs, CD39^+^ Tregs and CD39^neg^ Tregs, although any three memory CD4^+^ T‐cell populations able to be isolated by cell surface markers could potentially be tracked. These cells were labeled with three distinct fluorescent cell proliferation dyes (CFSE, CellTrace Violet and Cell Proliferation Dye eF670) and then all autologous PBMCs were reconstituted maintaining *ex vivo* cell ratios and CD25/OX40 AIM assays performed with CMV and HSV antigens. This approach enabled tracking of pre‐defined cell populations within antigen stimulated responses using both activation marker and cell proliferation readouts. We confirmed that although CD39^+^ Tregs comprise a substantial proportion of AIM assay responses, they do not make substantial contributions to the proliferative response. This extends the utility of AIM assays to enable parallel analysis of the relative contribution of several CD4^+^ memory T‐cell subsets to recall responses.

## Graphical abstract

Activation‐induced marker (AIM) assays enable rapid detection of antigen‐specific T cells. However, cellular activation changes cell phenotypic marker expression preventing the determination of cell subsets within these responses. This approach allows up to three unique cell populations to be tracked within AIM assays and subsequent proliferation assays.

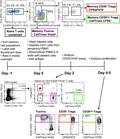

## INTRODUCTION

Assays to study antigen‐specific CD4^+^ T‐cell populations are in high demand, particularly those that do not require *a priori* knowledge of the immunogenic epitopes or their HLA restriction elements. In this regard, a broad group of assays, termed activation induced marker (AIM) assays, have proven to be extremely useful and have been used widely to analyze the T‐cell response to various disease antigens, most recently SARS‐CoV‐2, following infection and vaccination.^1^, ^2^, ^3^ One such assay that was developed in our laboratory uses activation induced co‐expression of CD25 (IL‐2Rα) and OX40 (CD134) after 44–48 h incubation with antigen to define the antigen‐specific CD4^+^ T cells.^4^ This CD25/OX40 assay can use either heparinized peripheral blood or isolated peripheral blood mononuclear cells (PBMCs), is not affected by bystander activation or cell death, shows strong correlation with cytokine release assays and tetramer staining and has been used to study recall responses to viral, bacterial, mycobacterial, fungal, food, vaccine and self‐antigens.^4^, ^5^, ^6^, ^7^, ^8^, ^9^, ^10^ Importantly, the CD25/OX40 assay detects CD4^+^ T cells from diverse functional lineages with specificity for the recall antigen used,^1^ including T follicular helper (Tfh) cells^2^ and FOXP3^+^ regulatory T cells (Tregs).^9^ Tregs are notoriously difficult to identify following activation, as their defining markers of constitutively high levels of CD25 and low levels of CD127 prior to stimulation are acquired by non‐Treg cells following T‐cell receptor activation.^11^ We have shown that CD39 expression is not affected within the 44–48 h time course of this assay and can be used in combination with CD25 and OX40 to identify a population of cells highly enriched (> 85%) for Tregs.^9^ We describe here a method to track three cell populations concurrently within antigen‐specific responses (CD25/OX40 assay) and cell proliferative responses. This extends the utility of the CD25/OX40 assay, avoids issues with activation‐induced changes to CD4^+^ T‐cell lineage marker expression and can be readily used with other AIM and/or cytokine staining assays.

## RESULTS AND DISCUSSION

### Combination of three CFSE‐like fluorescent dyes to track antigen‐stimulated proliferation of sorted memory T cells

To track multiple cell populations within antigen‐specific responses using AIM and cell proliferation assays, we first investigated a range of cell proliferation dyes that met the following criteria: (1) they labeled cells using the same mechanism; (2) they had minimal toxicity; (3) they had minimal crossover staining in co‐culture; (4) they could be used in conjunction with fluorescent mAbs used to analyze the CD25/OX40 assay and (5) they could be used to label very small numbers of cells (2–5 × 10^4^ cells). Previous work by Quah and Parish^12^ demonstrated that CellTrace Violet (CTV), carboxyfluorescein succinimidyl ester (CFSE) and Cell Proliferation Dye eFluor 670 (CPDeF670) could be used in co‐culture assay with minimal toxicity and crossover staining and tracked cell proliferation equally well, therefore these reagents were selected and optimized for use in this assay.

**Figure 1:**
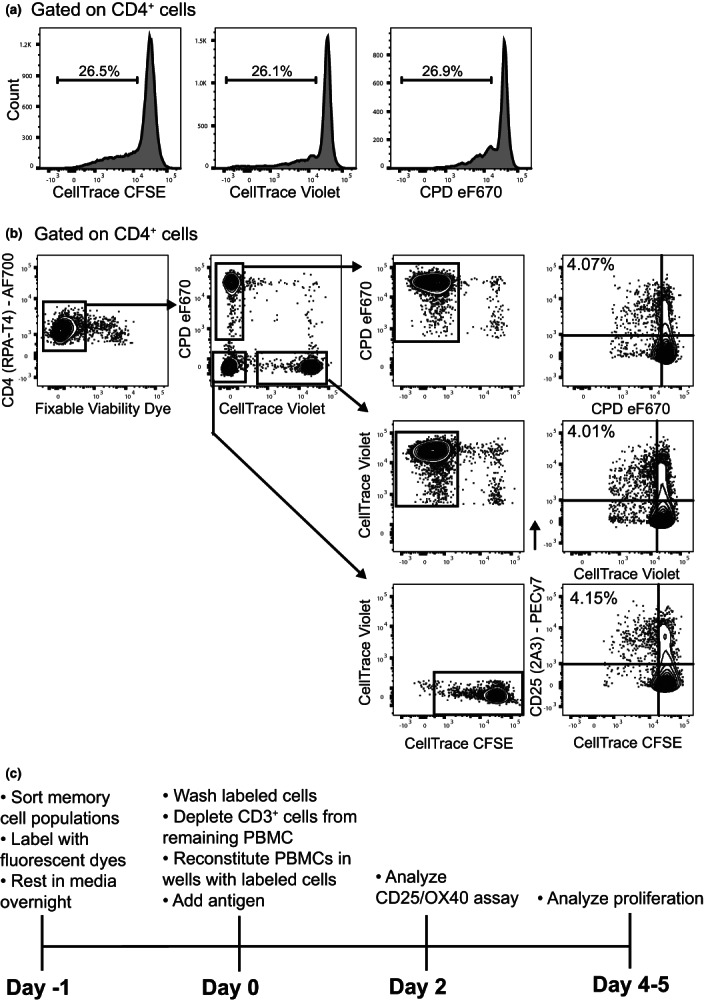
Comparative proliferation of CFSE, CTV and CPDeF670 labeled cells. **(a)** Unstimulated *ex vivo* CD4^+^CD45RO^+^CD25^high^CD127^low^ Tconv cells were sorted and stained with either CTV, CFSE or CPDeF670 and stimulated in separate wells for 4 days with soluble anti‐CD3 in the presence of autologous CD3‐depleted PBMCs, representative plots for *n* = 2 donors from independent experiments. **(b)** Labeled Tconv cells from a CMV seropositive donor were combined and stimulated with CMV lysate in the presence of autologous CD3‐depleted PBMCs for 5 days. Gating strategy for analyzing cell proliferation within individual dye‐labeled populations. Data are representative of *n* = 2 donors from independent experiments. **(c)** Schematic of assay setup and analysis timeline.

To confirm that CTV, CFSE and CPDeF670 were equivalent in their detection of cell proliferation, we first performed optimization experiments with sorted memory conventional T cells (Tconv; CD4^+^CD45RO^+^CD127^+^CD25^low^; mAb panel in Table 1). Of note, we used sorted memory cell populations in this study as it has been shown previously that antigen‐specific responses detected in the CD25/OX40 assay arise almost entirely from memory CD4^+^CD45RO^+^ T‐cell populations.^13^ The cells were labeled with one of the dyes and then each population was stimulated in separate wells for 4 days with soluble anti‐CD3 and autologous CD3‐depleted PBMCs as a source of antigen presenting cells. We observed equivalent proliferation for all three dyes, as described previously^12^ (Figure 1a, average %CV = 4.29). We next established co‐cultures with all three labeled cell populations from a cytomegalovirus (CMV) seropositive donor and stimulated with CMV lysate in the presence of autologous CD3‐depleted PBMCs for 4 or 5 days. The gating strategy used to identify each labeled cell population is shown in Figure 1b. Viable CD4^+^ T cells were gated, then the cells were separated into: CTV^+^, CPDeF670^+^ and CTV^neg^CPDeF670^neg^ (containing CFSE^+^ cells). Any CFSE^+^ cells within CTV^+^ and CPD^+^ cell populations were excluded and CFSE^+^ cells were gated (Figure 1b). This strategy enabled separate analysis of the three labeled populations and confirmed the amount of cell proliferation in each proliferation dye labeled population was equivalent for CMV lysate stimulation (Figure 1b). The optimized assay setup and analysis timeline is shown in Figure 1c.

**Table 1:** Fluorescent mAb panels

Target	Clone	Conjugate	Amount	Company
***Ex vivo*** **sorting panel** ^a^				
CD3	SK7	PerCPCy5.5	1/200	BD
CD4	RPA‐T4	AlexaFluor®700	1/100	BD
CD45RO	UCHL1	ECD	1/100	Beckman Coulter
CD25	2A3	APC	1/40	BD
CD127	eBioRDR5	eFluor®450	1/50	eBioscience
CD39	A1	PECy7	1/50	eBioscience
**CD25/OX40 assay panel** ^a^				
CD3	SK7	PerCPCy5.5	1/200	BD
CD4	RPA‐T4	AlexaFluor®700	1/100	BD
CD25	2A3	PECy5	1/40	BD
OX40	L106	PE	1/10	BD
CD39	A1	PECy7	1/50	eBioscience
Live/Dead near IR stain			1/1000	ThermoFisher Scientific
CellTrace Violet				ThermoFisher Scientific
CellTrace CFSE				ThermoFisher Scientific
Cell Proliferation Dye eFluor670				eBioscience
**Proliferation assay panel**				
CD3	SK7	PerCPCy5.5	1/200	BD
CD4	RPA‐T4	AlexaFluor®700	1/100	BD
CD25	2A3	PECy7	1/40	BD
CD39	A1	PE	1/50	eBioscience
Live/Dead near IR stain			1/1000	ThermoFisher Scientific
CellTrace Violet				ThermoFisher Scientific
CellTrace CFSE				ThermoFisher Scientific
Cell Proliferation Dye eFluor670				eBioscience

a Cell sorting mAbs will still be present on cells for CD25/OX40 assay analysis 44–48 h post‐sort. Panels need to be compatible.

### High proportions of CD39 ^+^ Tregs within antigen‐stimulated early activation, but not proliferative, responses

We next used this cell proliferation dye‐labeling protocol to analyze the relative contributions of memory Tconv cells, CD39^+^ Tregs and CD39^neg^ Tregs to both the initial antigen‐specific response (using the CD25/OX40 assay) and subsequent antigen‐driven *in vitro* cell proliferation. These populations were chosen because, as previously stated, it is particularly difficult to identify Tregs following activation and we had previously identified that CD39^+^ Tregs comprised substantial proportions of CD25/OX40 assay responses. By using this approach, we aimed to confirm the relative proportions of CD39^+^ and CD39^neg^ Tregs, as defined by the gold standard CD127^low^CD25^high^ gating strategy prior to cell activation, within assays measuring antigen‐driven activation (after 44 h) and proliferation (after 4 or 5 days).

**Figure 2:**
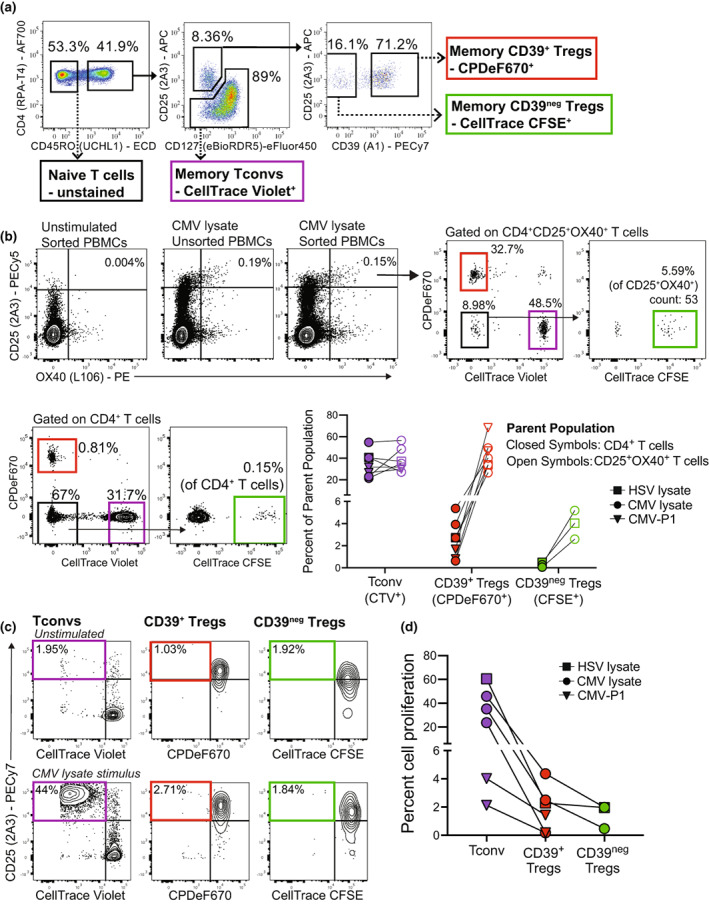
Isolation and proliferation tracking of *ex vivo* memory T‐cell populations. **(a)** Gating strategy for isolating unstimulated *ex vivo* CD4^+^ naïve T cells (CD45RO^neg^), memory Tconvs (CD45RO^+^CD25^low^CD127^+^, labeled with CTV); memory Tregs (CD45RO^+^CD25^high^CD127^low^) that were CD39^neg^ (labeled with CFSE); and CD39^+^ (labeled with CPDeF670). **(b)** Representative responses for CMV lysate stimulated or unstimulated wells with labeled, sorted PBMCs or unsorted PBMCs. Proportions of dye‐labeled memory populations within total CD4^+^ T cells and antigen‐specific CD25^+^OX40^+^ cells for assays stimulated with HSV lysate (*n* = 1, square symbols), CMV‐lysate (*n* = 4, circle symbols) or CMV‐P1 (*n* = 2, triangle symbols), all independent experiments. Due to low cell numbers, CFSE^+^ CD39^neg^ Tregs were quantified for *n* = 1 HSV lysate and *n* = 2 CMV lysate. **(c)** Representative FACS plots showing how, after gating as per Figure 1b, proliferating cells were defined as CD25^+^dye^dim^, with quadrants set using unstimulated cells. **(d)** Cell proliferation from assays stimulated with either HSV lysate (*n* = 1), CMV lysate (*n* = 3) or CMV‐P1 (*n* = 2), all independent experiments.

*Ex vivo* PBMCs were first separated by cell sorting into CD4^+^CD45RO^neg^ naïve T cells and CD4^+^CD45RO^+^ memory T cells. Memory T cells were further divided into CD127^+^CD25^low^ Tconv cells (labeled with CTV) and CD127^low^CD25^high^CD39^+^ Tregs (labeled with CPDeF670) and CD127^low^CD25^high^CD39^neg^ Tregs (labeled with CFSE; Figure 2a). Autologous assays were set up in 6‐well plates or 25 cm^2^ flasks that contained all three stained populations with unlabeled naïve CD4^+^ T cells and unlabeled CD3‐depleted PBMCs as a source of antigen presenting cells. All CD4^+^ T‐cell populations were added back to retain the same cell proportions as in *ex vivo* PBMCs, with CD3‐depleted PBMCs added at 1/6^th^ the number of CD4^+^ T cells. Co‐cultures were either left unstimulated (negative control) or stimulated with antigen. To enable matched analysis of labeled T‐cell proportions within responses at 44 h and after 4 or 5 days, we only collected a portion of assay wells at 44 h for analysis and the remainder was left in incubator.

After 44 h, being a late pre‐proliferation time point, using the mAb panel in Table 1 and fluorescent dye gating strategy in Figure 1b, we could detect all three labeled cell populations within total live CD4^+^ T cells at the same ratios initially added. Representative plots are shown in Figure 2b for a CMV lysate stimulated assay. As the amounts of CFSE^+^CD39^neg^ Tregs were very low, and often below the level of accurate quantification, we performed additional experiments with just CTV labeled Tconv and CPDeF670 labeled CD39^+^ Tregs. Within the total CD4^+^ T cells in reconstituted wells, CTV^+^ Tconv cells were the most abundant, comprising an average of 34% of CD4^+^ T cells (*n* = 7) with CPDeF670 labeled CD39^+^ Tregs an average 2.5% (*n* = 7) and CFSE^+^CD39^neg^ Tregs an average 0.28% (*n* = 5; Figure 2b).

We next assessed whether this complex cell manipulation was affecting the reliability of the CD25/OX40 assay through inducing non‐specific cell activation or reducing antigen‐specific responses. We confirmed that in wells with reconstituted labeled cells left unstimulated, at 44 h there was < 0.02% background (CD25^+^OX40^+^ cells as a percentage of CD4^+^ T cells; *n* = 7), which is below the assay cutoff (Figure 2b). We next compared responses from wells with reconstituted labeled cells with the wells containing total unsorted PBMCs from the same donor. For *n* = 6 donors stimulated with Herpes Simplex Virus‐1 (HSV) lysate (*n* = 1), CMV lysate (*n* = 4) or a 15mer peptide from CMV pp65 (CMV‐P1; *n* = 1), the reconstituted wells had an average 15% loss of the proportion of detectable antigen‐specific CD4^+^CD25^+^OX40^+^ T cells (Figure 2b). This is potentially due to T‐cell loss through sorting and assay setup or due to changes in the composition and/or relative numbers of antigen presenting cells. However, this reduced response was still of sufficient magnitude to enable the analysis of constituent labeled cell populations and is similar to the reduced CD25/OX40 assay responses to protein antigens in PBMCs *vs*. whole blood, thought to be due to the loss of antigen presenting cells (unpublished data). Therefore, we recommend that parallel OX40 assays with unsorted PBMCs (or ideally fresh whole blood) are used to quantify accurately the circulating proportions of total antigen‐specific CD4^+^ T cells.

Importantly, and as we have shown previously,^9^ the less abundant CPDeF670^+^CD39^+^ Tregs comprised a substantial proportion (average 43%, *n* = 7; Figure 2b) of the antigen‐specific CD25^+^OX40^+^ cells, while CFSE^+^CD39^neg^ Tregs made negligible contributions (average 4%, *n* = 3). In contrast, the CTV^+^ Tconvs comprised a similar proportion of CD25^+^OX40^+^ cells (average 38%, *n* = 7) as seen within total CD4^+^ T cells. These findings were consistent for assays stimulated with either CMV lysate, CMV‐P1 or HSV lysate, showing the robustness of this approach. These data confirm that this methodology is suitable for determining the contribution of pre‐labeled memory T‐cell populations to antigen‐specific recall responses following 44 h antigen stimulation.

We next analyzed the proliferation of each dye‐labeled cell population by staining the remainder of the assay at day 4 or day 5 with the proliferation mAb panel and quantifying the CD25^+^dye^dim^ cells (Table 1, Figure 2c). Note that the time chosen for proliferation readout needs to be optimized for each antigen stimulation, so that the proliferating labeled cells (proliferation dye^dim^) can still be defined separately from the unlabeled cells.

It was consistently observed that the proliferative response was almost entirely composed of Tconvs, with negligible cell proliferation occurring within memory Treg populations (*n* = 6 donors using HSV lysate, CMV lysate or CMV‐P1 stimulus; Figure 2d). These data concur with previous data showing human Tregs derived from peripheral blood are hypo‐proliferative *in vitro*.^14^ However, whilst peripheral blood Tregs are largely hypo‐proliferative, Tregs from secondary lymphoid organs have been shown to secrete IL‐2 and have high proliferative potential *in vitro* ^14^ and varicella zoster virus‐specific Tregs proliferated to the same extent *in vivo* as memory effector T cells following antigen challenge.^15^ Therefore, although the observation that Tregs from peripheral blood are hypo‐proliferative *in vitro* is consistent with previous *in vitro* data, these cells may have an increased proliferative capacity *in vivo*. However, our study is limited by a small number of blood donors and antigens and these findings should be replicated in a larger study with additional antigens, including bacterial and fungal.

## CONCLUSION

We have validated a unique method that uses three cell proliferation dyes to label *ex vivo* memory Tconv and Treg populations, enabling their tracking within antigen‐specific responses in CD25/OX40 AIM assays and proliferation assays. This solves the problem of being unable to quantify accurately in AIM assays those T‐cell subsets whose surface lineage markers are altered following activation. This is particularly problematic for CD25^+^CD127^low^ Tregs, as this phenotype is adopted by Tconv cells upon activation.

Our data show that CD39^+^ Tregs, while only comprising 2.5% of CD4^+^ T cells in the well, make up nearly half of the CD25/OX40 assay response to CMV and HSV antigens, confirming our earlier studies.^9^ Although Tregs are present at a nearly 1:1 ratio, their primary role in this effector phase does not appear to be the suppression of Tconv‐cell proliferation. We also show that until day 5 the Tregs survive, but do not contribute to the proliferative response, further evidencing how proliferative measures of antigen specificity do not account for Tregs. Further studies should focus on exploring what are likely to be the more important roles of Tregs within memory responses to pathogens, being immune modulatory and tissue protective functions.^16^

Our population tracking method enables the relative contribution of distinct cell populations to recall responses to be assessed in conjunction with an AIM assay, within 24–48 h, or to cell proliferation over 4–5 days. This provides a platform for systematically altering the composition of cellular memory to detect discrete effects of cell subsets and enables more precise studies of CD4^+^ memory T cells generated following infection or vaccination.

## METHODS

### Samples

Peripheral blood from healthy individuals was collected in sodium heparin vacutainers (Becton‐Dickinson (BD), Franklin Lakes, USA) and PBMCs extracted by density gradient centrifugation over Ficoll‐Paque (GE Healthcare, Buckinghamshire, UK). This study was approved by St Vincent's Hospital Human Research Ethics Committee, Sydney, Australia (Ethics ID: HREC/10/SVH/130), and all subjects gave written informed consent.

### Reagents

Cytomegalovirus grade III antigen (Meridian Life Sciences, Memphis, USA), a lysate of purified CMV antigens from the AD169 CMV strain, was used at 2 μg mL^−1^ and Herpes Simplex Virus‐1 lysate (HSV; BioWhittaker, Walkersville, USA) was used at 1 μg mL^−1^. A previously described HLA‐DR*15 restricted CMV pp65 epitope CMV‐P1 (5′–LLQTGIHVRVSQPSL–3′) was synthesized to > 95% purity (Mimotopes, Mulgrave, Australia) and used at a final concentration of 10 μg mL^−1^.^9^

### Fluorescent mAb staining

Cells were stained for extracellular protein expression by incubation with fluorochrome conjugated mAbs in the dark at room temperature (RT) for 15 min. The Cells were then washed in phosphate buffered saline (PBS, ThermoFisher Scientific, Waltham, USA) supplemented with 0.1% bovine serum albumin (BSA, Sigma‐Aldrich, St Louis, USA) and centrifuged on a Beckman Coulter (Brea, USA) Allegra X‐15R centrifuged for 7 min at 335 *g* before being fixed with 0.5% paraformaldehyde (PFA; ProSciTech, Kirwan, Australia) in PBS and data acquired within 1 h.

### Cell sorting

Cell sorting was performed using a three‐laser FACS Aria II cell sorter (BD). Defined cell populations were isolated at > 90% purity. Unstimulated cell populations were sorted using a 70 μm nozzle. To optimize the recovery of small cell populations, the flow rate was adjusted to ensure a sorting efficiency of > 80%, resulting in a purity of > 95% for the sorted populations. The cells were sorted into sterile 5 mL polystyrene tubes (Invitrogen) containing fetal bovine serum (FBS, Bovogen Biopharmaceuticals, Keilor East, Australia) at RT.

### Fluorescent cell labeling

The cells were labeled with either CellTrace Violet (CTV), CellTrace CFSE (CFSE; ThermoFisher Scientific) or Cell Proliferation Dye eFluor 670 (CPDeF670; eBioscience, San Diego, USA) using 5 μM dye concentrations. For labeling with CTV, the cells were washed once in PBS and resuspended at 1 × 10^6^ cells mL^−1^ in PBS. Dye aliquots were stored at −80°C, thawed and diluted to working concentration in PBS immediately prior to use and discarded after one freeze/thaw. CTV labeling was performed by incubating cells at 1 × 10^6^ cells mL^−1^ for 20 min with 5 μM of dye in the dark at room temperature before washing once in complete media (RPMI‐1640 media with 10% human AB serum (Lonza, Basel, Switzerland), 1% L‐glutamine and 1% penicillin/streptomycin (ThermoFisher Scientific)) by centrifugation at 335 *g*. CFSE and CPDeF670 labeling was performed by incubating the cells at 1 × 10^6^ cells mL^−1^ in complete media for 5 min with 5 μM of dye before washing three times with RPMI‐1640 supplemented with 10% FBS. Labeled cells were incubated separately overnight in complete media to enable cellular release of any unincorporated dye into the supernatant. The cells were then washed once in complete media before being utilized in cell culture experiments. Analysis of cell viability was performed using Live/Dead near IR fixable staining kit (ThermoFisher Scientific) according to the manufacturer's instructions.

### Tracking cell populations within the CD25/OX40 AIM assay

Populations of interest were first isolated from unstimulated PBMCs by cell sorting (4‐way sort), then labeled with either CFSE, CTV or CPDeF670. Unlabeled autologous PBMCs were depleted of CD3^+^ T cells using CD3 Dynabeads according to the manufacturer's instructions (ThermoFisher Scientific) and were used as a source of antigen presenting cells. The cell culture density was 1.5–2 × 10^6^ cells mL^−1^ containing (1) CTV^+^ population, (2) CPDeF670^+^ population, (3) CFSE^+^ population, (4) unlabeled naïve CD4^+^ T cells and (5) unlabeled CD3‐depleted autologous PBMCs. This PBMC reconstitution was calculated to maintain the *ex vivo* ratios of each cell population, with CD3^neg^ PBMCs added at ⅙ of the final cell concentration. For all CD25/OX40 population tracking AIM assays, following well reconstitution and antigen addition, the cultures were incubated at 37°C in 5% CO_2_ (humidified atmosphere). Assays were performed in 500 μL in 24‐well plates or in 5 mL in 25 cm^2^ flasks (Corning) in complete media. After a 44–48 h incubation with antigen, the cells were stained with the CD25/OX40 assay analysis mAb panel then analyzed by flow cytometry. If cell proliferation was being measured, then the cultures were incubated with antigen for 4 or 5 days and stained with the proliferation analysis mAb panel (mAb panels in Table 1).

### Flow cytometry data acquisition and analysis

Cells were analyzed on a four‐laser LSRII flow cytometer (BD), with a minimum of 100 000 events collected. Daily QC was done using cytometer setup and tracking beads (IAW instrument operator manual) and compensation was performed with unstained cells and cells stained with a single mAb per tube conjugated to fluorochromes used in experimental sample analysis. Analysis was performed using FlowJo software (BD), with gated cell populations only included in the analysis if they contained a minimum of 20 events to minimize stochastic variation.

## AUTHOR CONTRIBUTIONS

**Laura Cook:** Conceptualization; investigation; methodology; writing – original draft; writing – review and editing. **John Zaunders:** Conceptualization; methodology; supervision; writing – review and editing. **Nabila Seddiki:** Conceptualization; methodology; supervision; writing – review and editing. **David van Bockel:** Supervision; writing – review and editing. **Anthony D Kelleher:** Conceptualization; funding acquisition; resources; supervision; writing – review and editing. **C Mee Ling Munier:** Conceptualization; methodology; supervision; writing – review and editing.

## CONFLICT OF INTEREST

The authors declare no conflicts of interest.

## Data Availability

The data that support the findings of this study are available from the corresponding author upon reasonable request.
